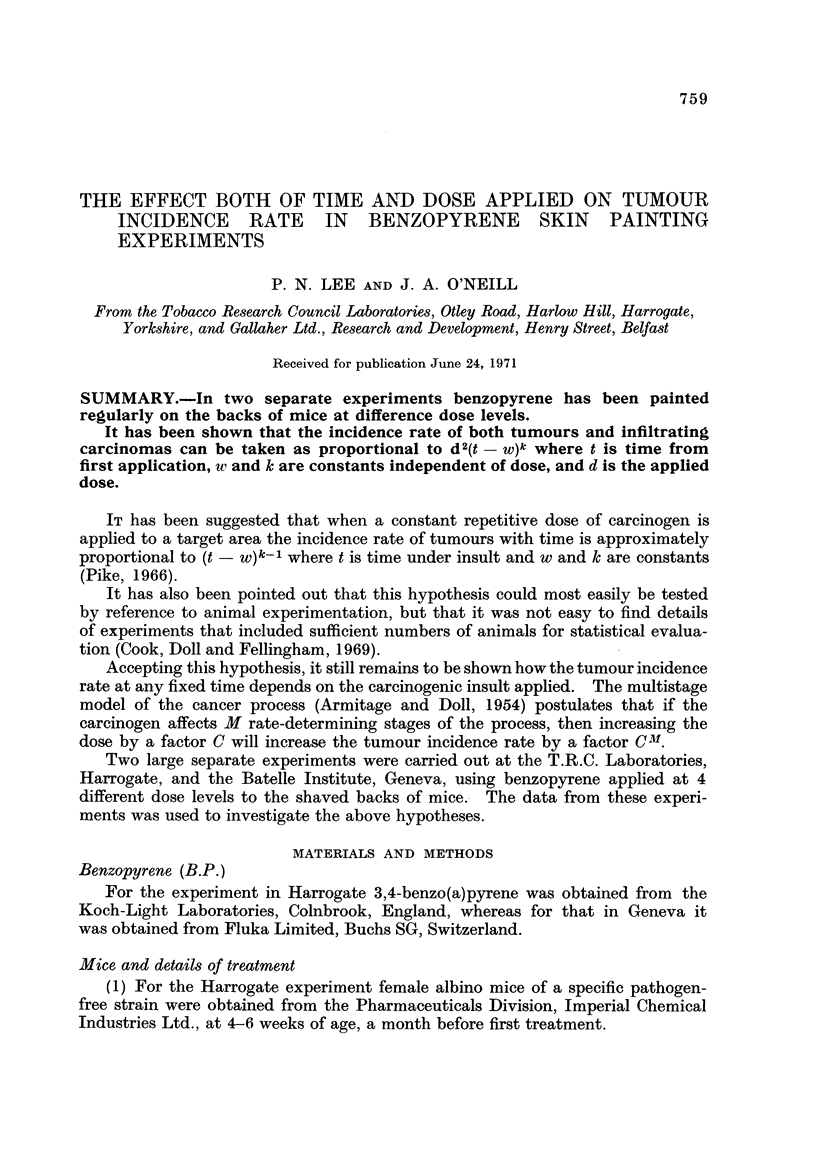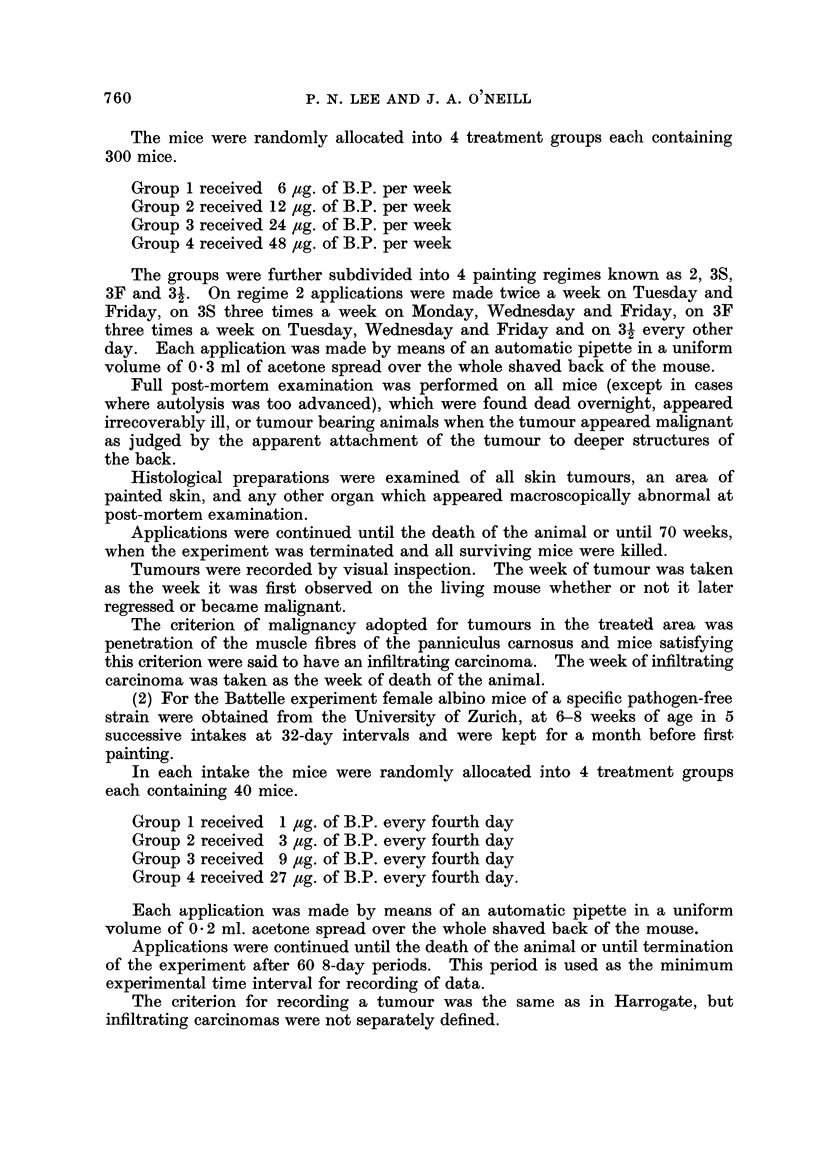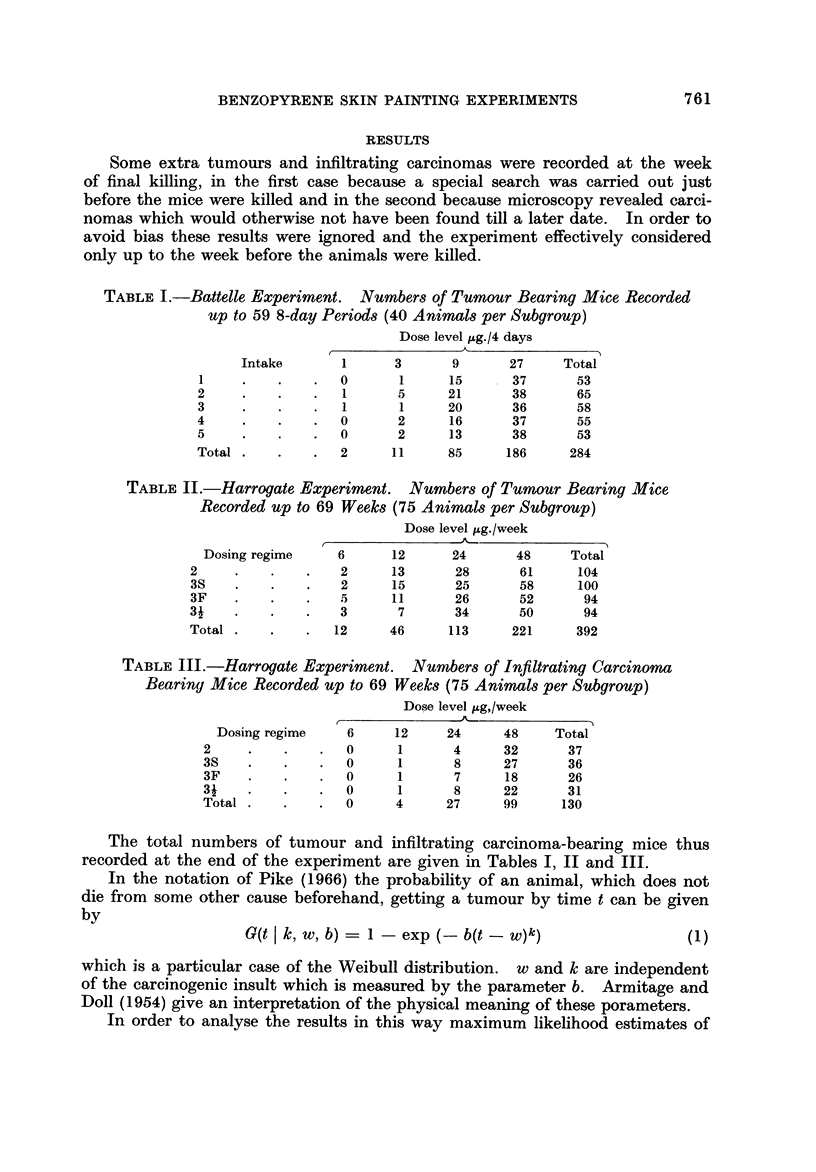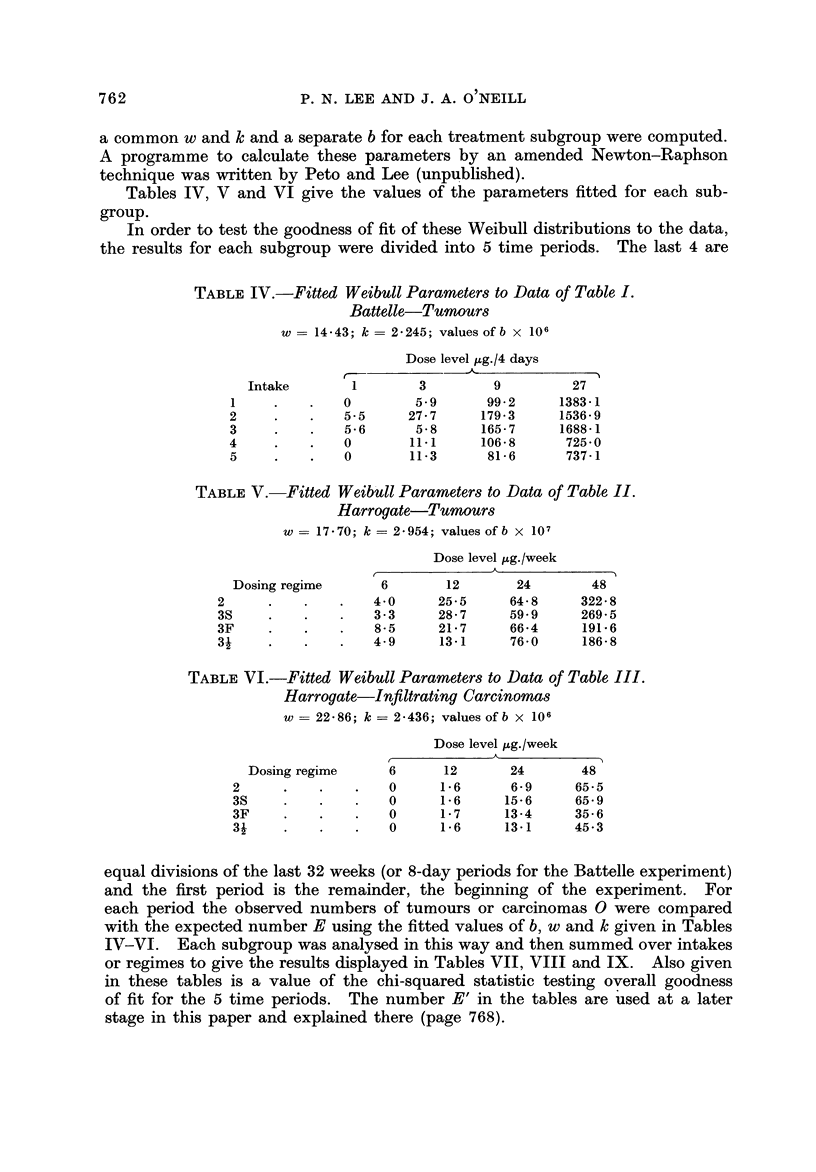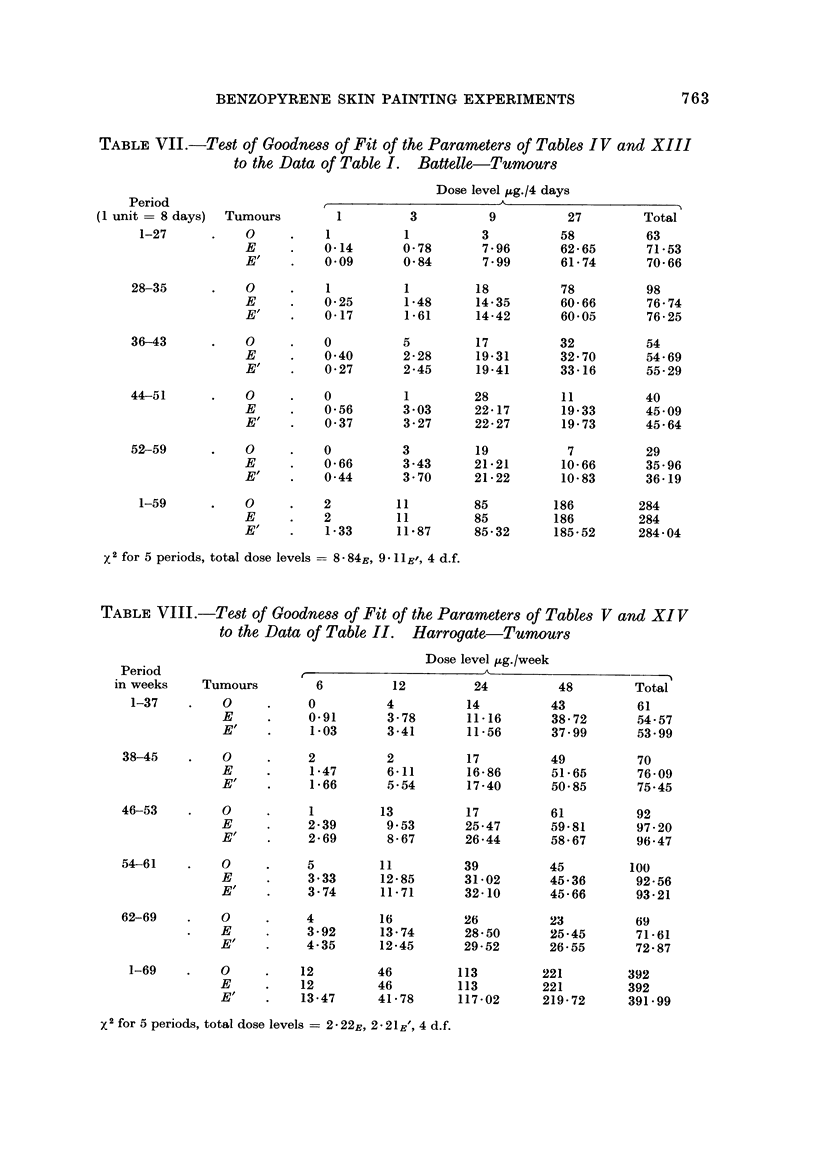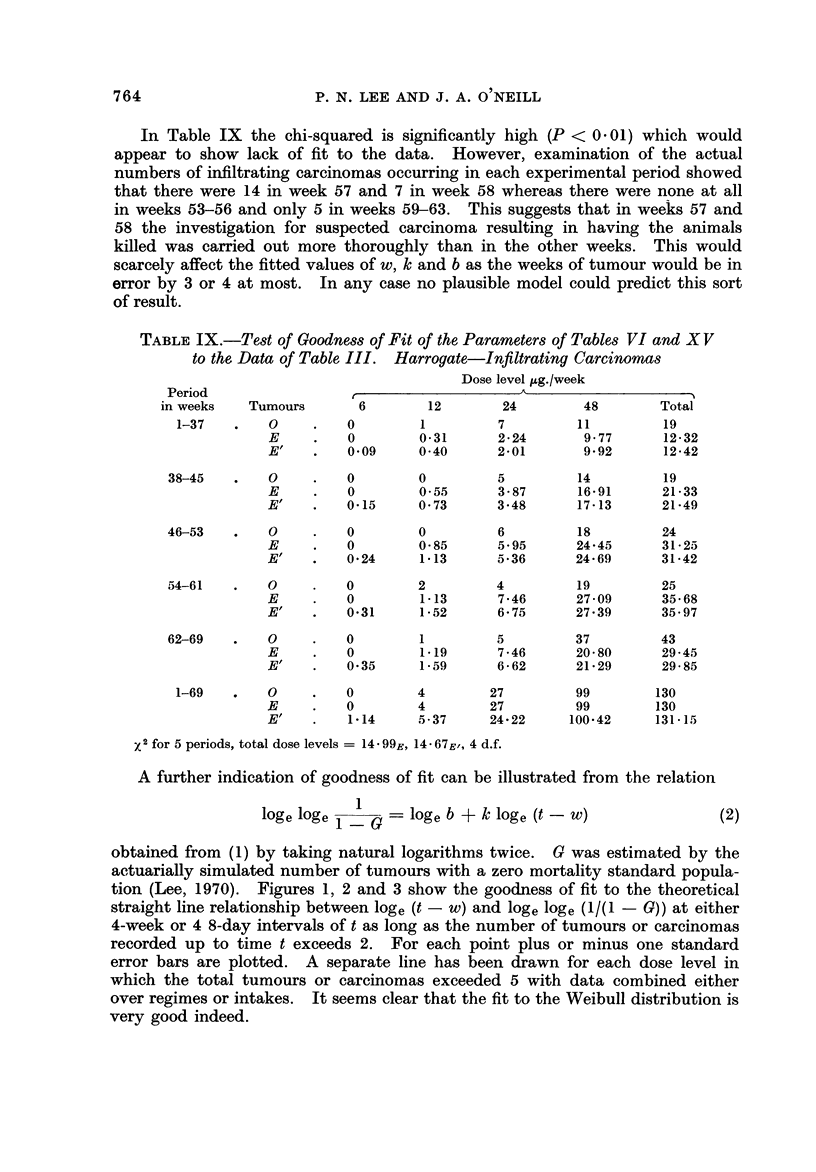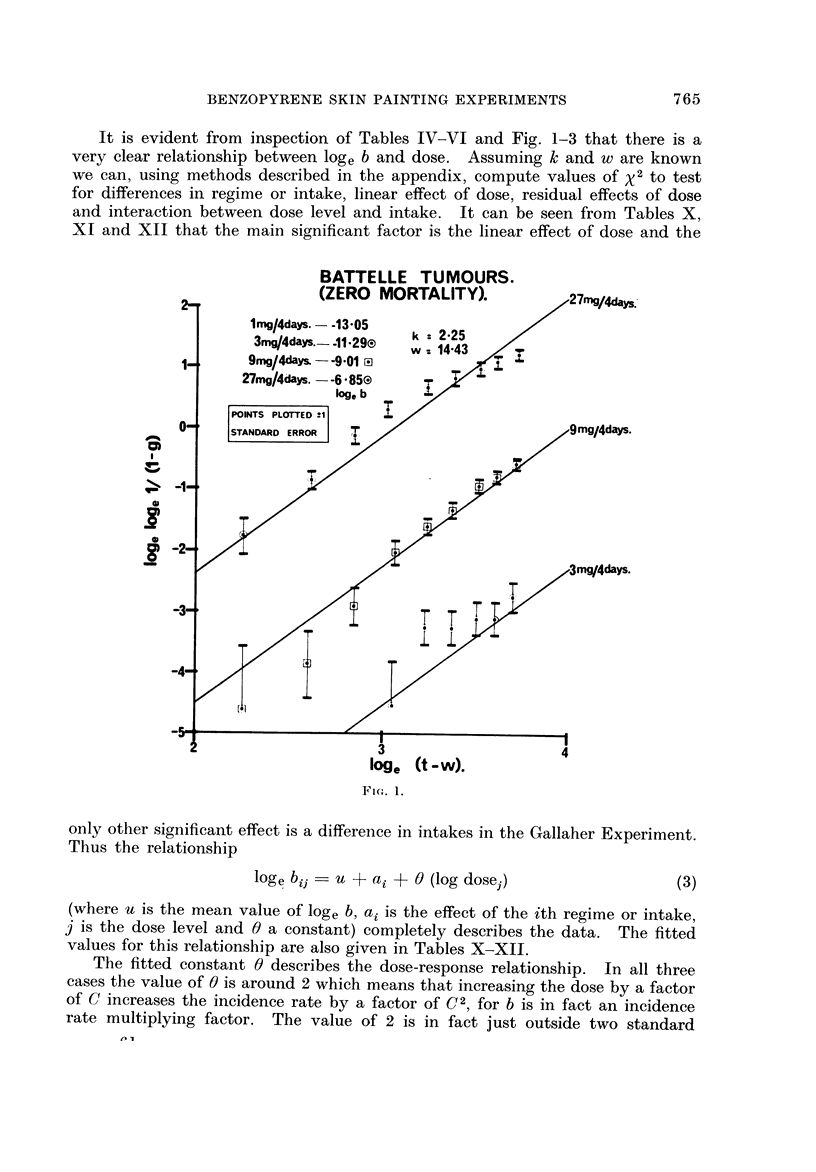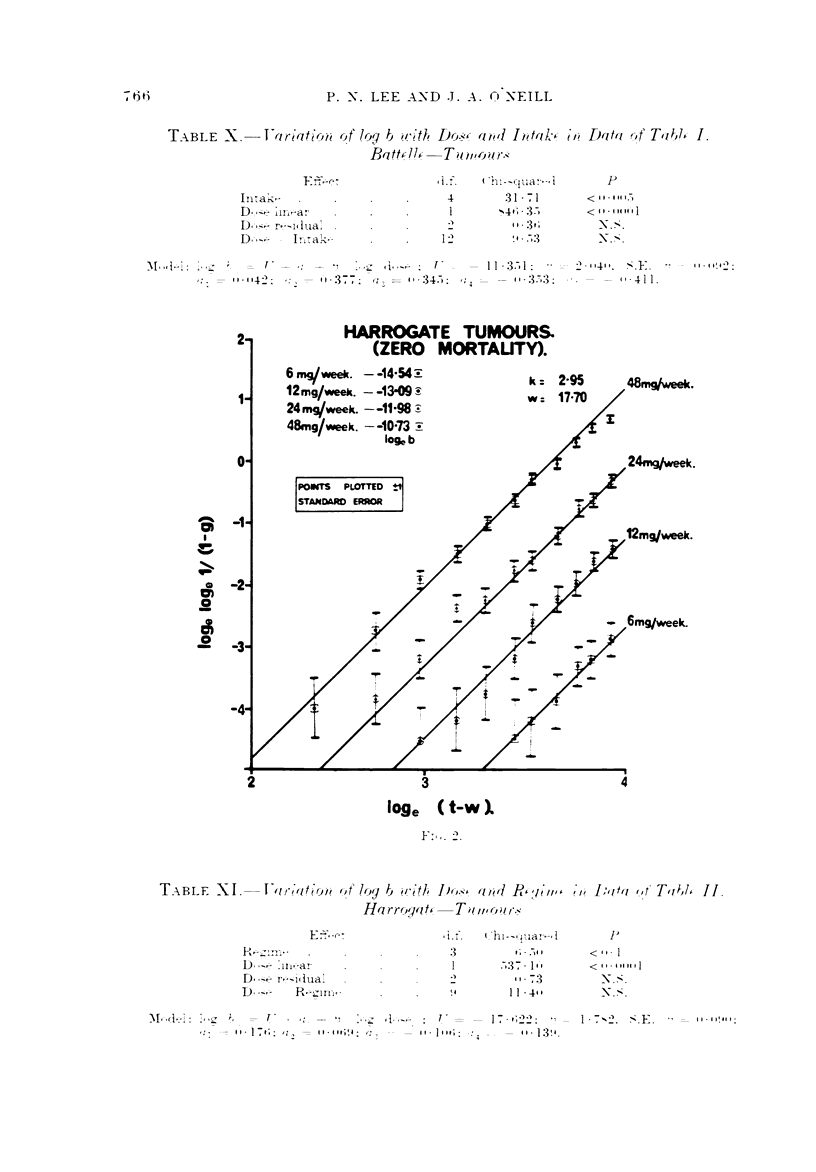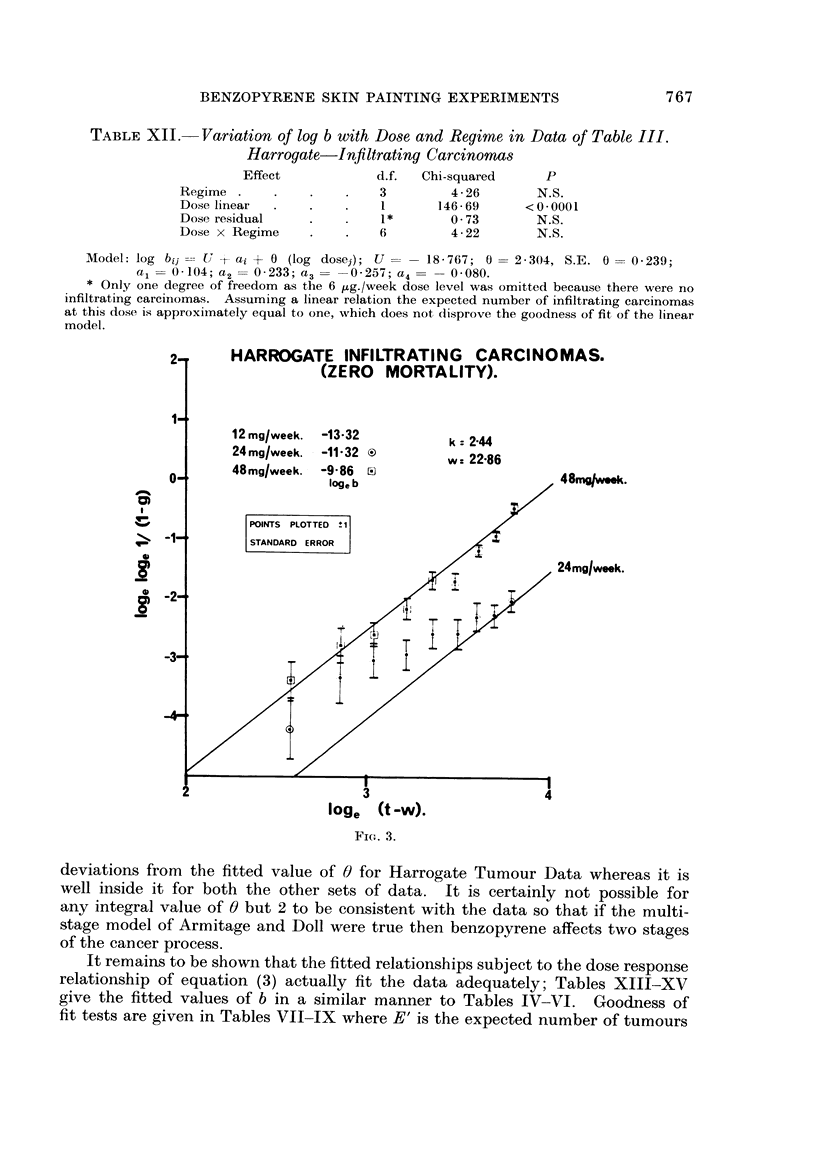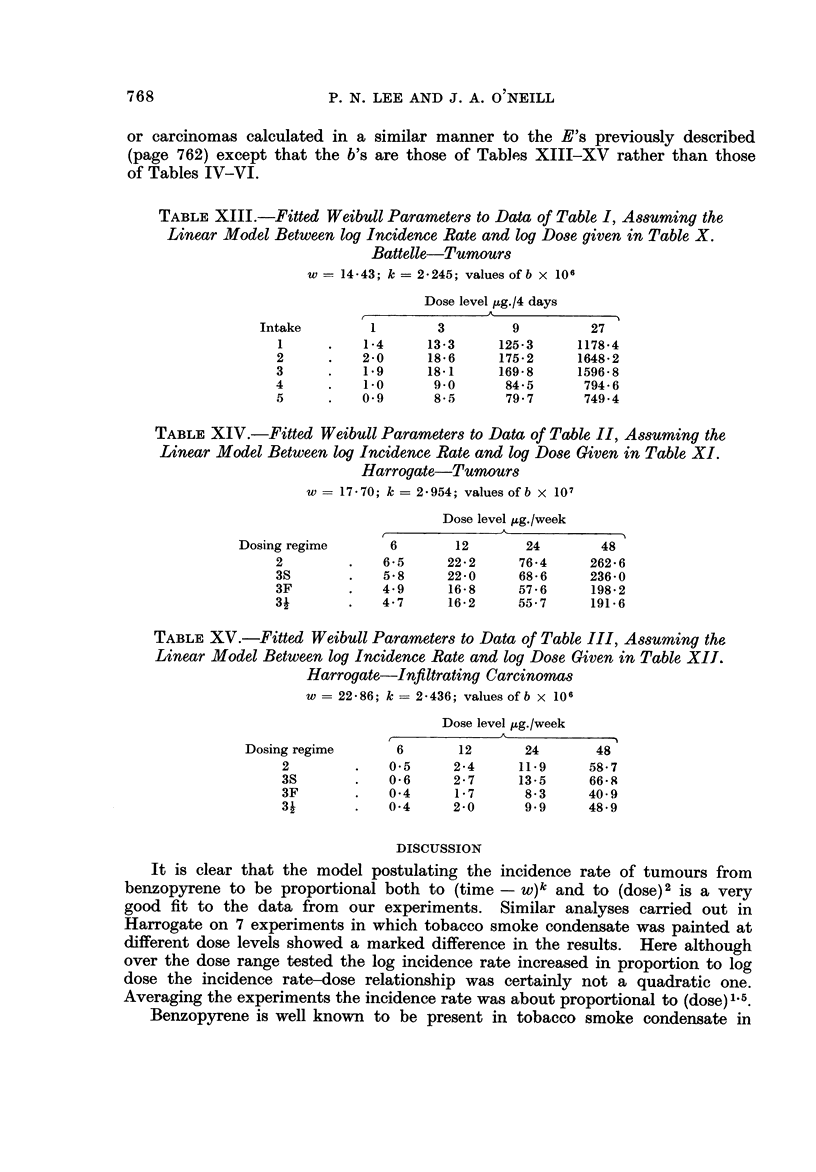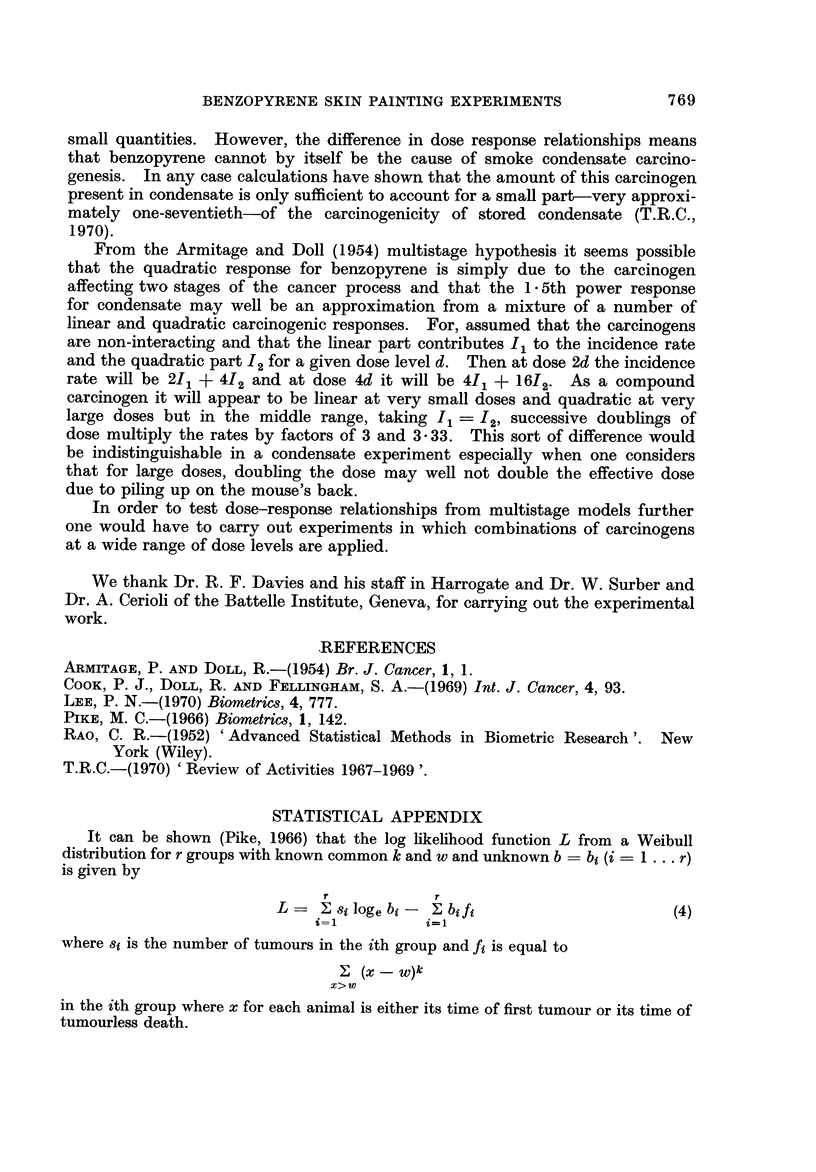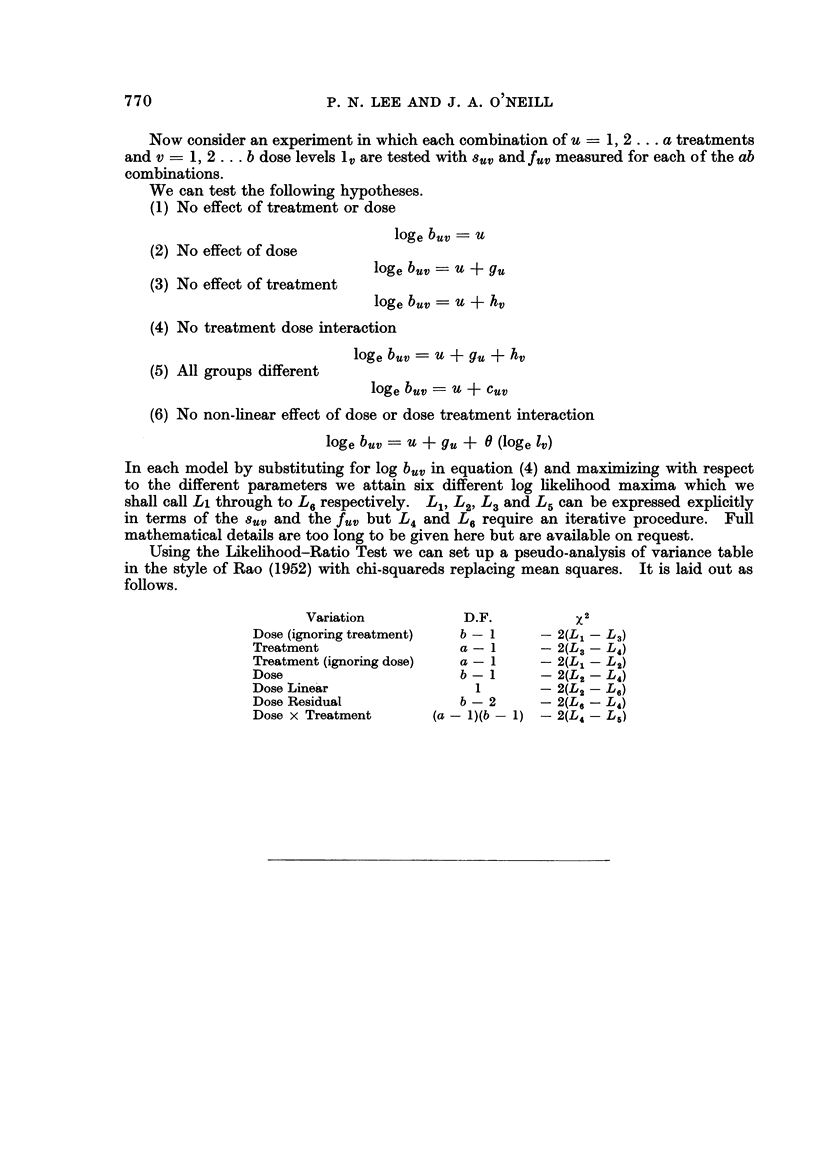# The Effect Both of Time and Dose Applied on Tumour Incidence Rate in Benzopyrene Skin Painting Experiments

**DOI:** 10.1038/bjc.1971.90

**Published:** 1971-12

**Authors:** P. N. Lee, J. A. O'Neill

## Abstract

In two separate experiments benzopyrene has been painted regularly on the backs of mice at difference dose levels.

It has been shown that the incidence rate of both tumours and infiltrating carcinomas can be taken as proportional to d^2^(*t — w*)^*k*^ where *t* is time from first application, *w* and *k* are constants independent of dose, and *d* is the applied dose.


### THE EFFECT BOTH OF TIME AND DOSE APPLIED ON TUMOUR INCIDENCE RATE IN BENZOPYRENE SKIN PAINTING EXPERIMENTS

P. N. LEE AND J. A. O'NEILL

*From the Tobacco Research Council Laboratories, Otley Road, Harlow Hill, Harrogate, Yorkshire, and Gallaher Ltd., Research and Development, Henry Street, Belfast*

Received for publication June 24, 1971

**SUMMARY.—In two separate experiments benzopyrene has been painted regularly on the backs of mice at difference dose levels.**

**It has been shown that the incidence rate of both tumours and infiltrating carcinomas can be taken as proportional to $d^2(t - w)^k$ where $t$ is time from first application, $w$ and $k$ are constants independent of dose, and $d$ is the applied dose.**

It has been suggested that when a constant repetitive dose of carcinogen is applied to a target area the incidence rate of tumours with time is approximately proportional to $(t - w)^{k-1}$ where $t$ is time under insult and $w$ and $k$ are constants (Pike, 1966).

It has also been pointed out that this hypothesis could most easily be tested by reference to animal experimentation, but that it was not easy to find details of experiments that included sufficient numbers of animals for statistical evaluation (Cook, Doll and Fellingham, 1969).

Accepting this hypothesis, it still remains to be shown how the tumour incidence rate at any fixed time depends on the carcinogenic insult applied. The multistage model of the cancer process (Armitage and Doll, 1954) postulates that if the carcinogen affects $M$ rate-determining stages of the process, then increasing the dose by a factor $C$ will increase the tumour incidence rate by a factor $C^M$.

Two large separate experiments were carried out at the T.R.C. Laboratories, Harrogate, and the Batelle Institute, Geneva, using benzopyrene applied at 4 different dose levels to the shaved backs of mice. The data from these experiments was used to investigate the above hypotheses.

### MATERIALS AND METHODS

### *Benzopyrene (B.P.)*

For the experiment in Harrogate 3,4-benzo(a)pyrene was obtained from the Koch-Light Laboratories, Colnbrook, England, whereas for that in Geneva it was obtained from Fluka Limited, Buchs SG, Switzerland.

### *Mice and details of treatment*

(1) For the Harrogate experiment female albino mice of a specific pathogen-free strain were obtained from the Pharmaceuticals Division, Imperial Chemical Industries Ltd., at 4–6 weeks of age, a month before first treatment.

The mice were randomly allocated into 4 treatment groups each containing 300 mice.

Group 1 received 6 $\mu$g. of B.P. per week
Group 2 received 12 $\mu$g. of B.P. per week
Group 3 received 24 $\mu$g. of B.P. per week
Group 4 received 48 $\mu$g. of B.P. per week

The groups were further subdivided into 4 painting regimes known as 2, 3S, 3F and $3\frac{1}{2}$. On regime 2 applications were made twice a week on Tuesday and Friday, on 3S three times a week on Monday, Wednesday and Friday, on 3F three times a week on Tuesday, Wednesday and Friday and on $3\frac{1}{2}$ every other day. Each application was made by means of an automatic pipette in a uniform volume of 0·3 ml of acetone spread over the whole shaved back of the mouse.

Full post-mortem examination was performed on all mice (except in cases where autolysis was too advanced), which were found dead overnight, appeared irrecoverably ill, or tumour bearing animals when the tumour appeared malignant as judged by the apparent attachment of the tumour to deeper structures of the back.

Histological preparations were examined of all skin tumours, an area of painted skin, and any other organ which appeared macroscopically abnormal at post-mortem examination.

Applications were continued until the death of the animal or until 70 weeks, when the experiment was terminated and all surviving mice were killed.

Tumours were recorded by visual inspection. The week of tumour was taken as the week it was first observed on the living mouse whether or not it later regressed or became malignant.

The criterion of malignancy adopted for tumours in the treated area was penetration of the muscle fibres of the panniculus carnosus and mice satisfying this criterion were said to have an infiltrating carcinoma. The week of infiltrating carcinoma was taken as the week of death of the animal.

(2) For the Battelle experiment female albino mice of a specific pathogen-free strain were obtained from the University of Zurich, at 6–8 weeks of age in 5 successive intakes at 32-day intervals and were kept for a month before first painting.

In each intake the mice were randomly allocated into 4 treatment groups each containing 40 mice.

Group 1 received 1 $\mu$g. of B.P. every fourth day

Group 2 received 3 $\mu$g. of B.P. every fourth day

Group 3 received 9 $\mu$g. of B.P. every fourth day

Group 4 received 27 $\mu$g. of B.P. every fourth day.

Each application was made by means of an automatic pipette in a uniform volume of 0·2 ml. acetone spread over the whole shaved back of the mouse.

Applications were continued until the death of the animal or until termination of the experiment after 60 8-day periods. This period is used as the minimum experimental time interval for recording of data.

The criterion for recording a tumour was the same as in Harrogate, but infiltrating carcinomas were not separately defined.

### RESULTS

Some extra tumours and infiltrating carcinomas were recorded at the week of final killing, in the first case because a special search was carried out just before the mice were killed and in the second because microscopy revealed carcinomas which would otherwise not have been found till a later date. In order to avoid bias these results were ignored and the experiment effectively considered only up to the week before the animals were killed.

TABLE I.—*Battelle Experiment. Numbers of Tumour Bearing Mice Recorded up to 59 8-day Periods (40 Animals per Subgroup)*

| | Dose level μg./4 days | | | | |
|---|---|---|---|---|---|
| Intake | 1 | 3 | 9 | 27 | Total |
| 1 . . . | 0 | 1 | 15 | 37 | 53 |
| 2 . . . | 1 | 5 | 21 | 38 | 65 |
| 3 . . . | 1 | 1 | 20 | 36 | 58 |
| 4 . . . | 0 | 2 | 16 | 37 | 55 |
| 5 . . . | 0 | 2 | 13 | 38 | 53 |
| Total . . . | 2 | 11 | 85 | 186 | 284 |

TABLE II.—*Harrogate Experiment. Numbers of Tumour Bearing Mice Recorded up to 69 Weeks (75 Animals per Subgroup)*

| | Dose level μg./week | | | | |
|---|---|---|---|---|---|
| Dosing regime | 6 | 12 | 24 | 48 | Total |
| 2 . . . | 2 | 13 | 28 | 61 | 104 |
| 3S . . . | 2 | 15 | 25 | 58 | 100 |
| 3F . . . | 5 | 11 | 26 | 52 | 94 |
| 3½ . . . | 3 | 7 | 34 | 50 | 94 |
| Total . . . | 12 | 46 | 113 | 221 | 392 |

TABLE III.—*Harrogate Experiment. Numbers of Infiltrating Carcinoma Bearing Mice Recorded up to 69 Weeks (75 Animals per Subgroup)*

| | Dose level μg,/week | | | | |
|---|---|---|---|---|---|
| Dosing regime | 6 | 12 | 24 | 48 | Total |
| 2 . . . | 0 | 1 | 4 | 32 | 37 |
| 3S . . . | 0 | 1 | 8 | 27 | 36 |
| 3F . . . | 0 | 1 | 7 | 18 | 26 |
| 3½ . . . | 0 | 1 | 8 | 22 | 31 |
| Total . . . | 0 | 4 | 27 | 99 | 130 |

The total numbers of tumour and infiltrating carcinoma-bearing mice thus recorded at the end of the experiment are given in Tables I, II and III.

In the notation of Pike (1966) the probability of an animal, which does not die from some other cause beforehand, getting a tumour by time $t$ can be given by

$$G(t \mid k, w, b) = 1 - \exp(-b(t - w)^k) \quad (1)$$

which is a particular case of the Weibull distribution. $w$ and $k$ are independent of the carcinogenic insult which is measured by the parameter $b$. Armitage and Doll (1954) give an interpretation of the physical meaning of these porameters.

In order to analyse the results in this way maximum likelihood estimates of a common $w$ and $k$ and a separate $b$ for each treatment subgroup were computed. A programme to calculate these parameters by an amended Newton–Raphson technique was written by Peto and Lee (unpublished).

Tables IV, V and VI give the values of the parameters fitted for each subgroup.

In order to test the goodness of fit of these Weibull distributions to the data, the results for each subgroup were divided into 5 time periods. The last 4 are

TABLE IV.—*Fitted Weibull Parameters to Data of Table I. Battelle—Tumours*

$w = 14 \cdot 43$; $k = 2 \cdot 245$; values of $b \times 10^6$

| | Dose level μg./4 days | | | |
|---|---|---|---|---|
| Intake | 1 | 3 | 9 | 27 |
| 1 | 0 | 5·9 | 99·2 | 1383·1 |
| 2 | 5·5 | 27·7 | 179·3 | 1536·9 |
| 3 | 5·6 | 5·8 | 165·7 | 1688·1 |
| 4 | 0 | 11·1 | 106·8 | 725·0 |
| 5 | 0 | 11·3 | 81·6 | 737·1 |

TABLE V.—*Fitted Weibull Parameters to Data of Table II. Harrogate—Tumours*

$w = 17 \cdot 70$; $k = 2 \cdot 954$; values of $b \times 10^7$

| | Dose level μg./week | | | |
|---|---|---|---|---|
| Dosing regime | 6 | 12 | 24 | 48 |
| 2 | 4·0 | 25·5 | 64·8 | 322·8 |
| 3S | 3·3 | 28·7 | 59·9 | 269·5 |
| 3F | 8·5 | 21·7 | 66·4 | 191·6 |
| 3½ | 4·9 | 13·1 | 76·0 | 186·8 |

TABLE VI.—*Fitted Weibull Parameters to Data of Table III. Harrogate—Infiltrating Carcinomas*

$w = 22 \cdot 86$; $k = 2 \cdot 436$; values of $b \times 10^6$

| | Dose level μg./week | | | |
|---|---|---|---|---|
| Dosing regime | 6 | 12 | 24 | 48 |
| 2 | 0 | 1·6 | 6·9 | 65·5 |
| 3S | 0 | 1·6 | 15·6 | 65·9 |
| 3F | 0 | 1·7 | 13·4 | 35·6 |
| 3½ | 0 | 1·6 | 13·1 | 45·3 |

equal divisions of the last 32 weeks (or 8-day periods for the Battelle experiment) and the first period is the remainder, the beginning of the experiment. For each period the observed numbers of tumours or carcinomas $O$ were compared with the expected number $E$ using the fitted values of $b$, $w$ and $k$ given in Tables IV–VI. Each subgroup was analysed in this way and then summed over intakes or regimes to give the results displayed in Tables VII, VIII and IX. Also given in these tables is a value of the chi-squared statistic testing overall goodness of fit for the 5 time periods. The number $E'$ in the tables are used at a later stage in this paper and explained there (page 768).

TABLE VII.—*Test of Goodness of Fit of the Parameters of Tables IV and XIII to the Data of Table I. Battelle—Tumours*

| Period (1 unit = 8 days) | Tumours | Dose level μg./4 days 1 | 3 | 9 | 27 | Total |
|---|---|---|---|---|---|---|
| 1–27 | *O* | 1 | 1 | 3 | 58 | 63 |
| | *E* | 0·14 | 0·78 | 7·96 | 62·65 | 71·53 |
| | *E′* | 0·09 | 0·84 | 7·99 | 61·74 | 70·66 |
| 28–35 | *O* | 1 | 1 | 18 | 78 | 98 |
| | *E* | 0·25 | 1·48 | 14·35 | 60·66 | 76·74 |
| | *E′* | 0·17 | 1·61 | 14·42 | 60·05 | 76·25 |
| 36–43 | *O* | 0 | 5 | 17 | 32 | 54 |
| | *E* | 0·40 | 2·28 | 19·31 | 32·70 | 54·69 |
| | *E′* | 0·27 | 2·45 | 19·41 | 33·16 | 55·29 |
| 44–51 | *O* | 0 | 1 | 28 | 11 | 40 |
| | *E* | 0·56 | 3·03 | 22·17 | 19·33 | 45·09 |
| | *E′* | 0·37 | 3·27 | 22·27 | 19·73 | 45·64 |
| 52–59 | *O* | 0 | 3 | 19 | 7 | 29 |
| | *E* | 0·66 | 3·43 | 21·21 | 10·66 | 35·96 |
| | *E′* | 0·44 | 3·70 | 21·22 | 10·83 | 36·19 |
| 1–59 | *O* | 2 | 11 | 85 | 186 | 284 |
| | *E* | 2 | 11 | 85 | 186 | 284 |
| | *E′* | 1·33 | 11·87 | 85·32 | 185·52 | 284·04 |

$\chi^2$ for 5 periods, total dose levels = $8{\cdot}84_E$, $9{\cdot}11_{E'}$, 4 d.f.

TABLE VIII.—*Test of Goodness of Fit of the Parameters of Tables V and XIV to the Data of Table II. Harrogate—Tumours*

| Period in weeks | Tumours | Dose level μg./week 6 | 12 | 24 | 48 | Total |
|---|---|---|---|---|---|---|
| 1–37 | *O* | 0 | 4 | 14 | 43 | 61 |
| | *E* | 0·91 | 3·78 | 11·16 | 38·72 | 54·57 |
| | *E′* | 1·03 | 3·41 | 11·56 | 37·99 | 53·99 |
| 38–45 | *O* | 2 | 2 | 17 | 49 | 70 |
| | *E* | 1·47 | 6·11 | 16·86 | 51·65 | 76·09 |
| | *E′* | 1·66 | 5·54 | 17·40 | 50·85 | 75·45 |
| 46–53 | *O* | 1 | 13 | 17 | 61 | 92 |
| | *E* | 2·39 | 9·53 | 25·47 | 59·81 | 97·20 |
| | *E′* | 2·69 | 8·67 | 26·44 | 58·67 | 96·47 |
| 54–61 | *O* | 5 | 11 | 39 | 45 | 100 |
| | *E* | 3·33 | 12·85 | 31·02 | 45·36 | 92·56 |
| | *E′* | 3·74 | 11·71 | 32·10 | 45·66 | 93·21 |
| 62–69 | *O* | 4 | 16 | 26 | 23 | 69 |
| | *E* | 3·92 | 13·74 | 28·50 | 25·45 | 71·61 |
| | *E′* | 4·35 | 12·45 | 29·52 | 26·55 | 72·87 |
| 1–69 | *O* | 12 | 46 | 113 | 221 | 392 |
| | *E* | 12 | 46 | 113 | 221 | 392 |
| | *E′* | 13·47 | 41·78 | 117·02 | 219·72 | 391·99 |

$\chi^2$ for 5 periods, total dose levels = $2{\cdot}22_E$, $2{\cdot}21_{E'}$, 4 d.f.

In Table IX the chi-squared is significantly high ($P < 0 \cdot 01$) which would appear to show lack of fit to the data. However, examination of the actual numbers of infiltrating carcinomas occurring in each experimental period showed that there were 14 in week 57 and 7 in week 58 whereas there were none at all in weeks 53–56 and only 5 in weeks 59–63. This suggests that in weeks 57 and 58 the investigation for suspected carcinoma resulting in having the animals killed was carried out more thoroughly than in the other weeks. This would scarcely affect the fitted values of $w$, $k$ and $b$ as the weeks of tumour would be in error by 3 or 4 at most. In any case no plausible model could predict this sort of result.

TABLE IX.—*Test of Goodness of Fit of the Parameters of Tables VI and XV to the Data of Table III. Harrogate—Infiltrating Carcinomas*

| Period in weeks | Tumours | Dose level μg./week 6 | 12 | 24 | 48 | Total |
|---|---|---|---|---|---|---|
| 1–37 | *O* | 0 | 1 | 7 | 11 | 19 |
| | *E* | 0 | 0·31 | 2·24 | 9·77 | 12·32 |
| | *E'* | 0·09 | 0·40 | 2·01 | 9·92 | 12·42 |
| 38–45 | *O* | 0 | 0 | 5 | 14 | 19 |
| | *E* | 0 | 0·55 | 3·87 | 16·91 | 21·33 |
| | *E'* | 0·15 | 0·73 | 3·48 | 17·13 | 21·49 |
| 46–53 | *O* | 0 | 0 | 6 | 18 | 24 |
| | *E* | 0 | 0·85 | 5·95 | 24·45 | 31·25 |
| | *E'* | 0·24 | 1·13 | 5·36 | 24·69 | 31·42 |
| 54–61 | *O* | 0 | 2 | 4 | 19 | 25 |
| | *E* | 0 | 1·13 | 7·46 | 27·09 | 35·68 |
| | *E'* | 0·31 | 1·52 | 6·75 | 27·39 | 35·97 |
| 62–69 | *O* | 0 | 1 | 5 | 37 | 43 |
| | *E* | 0 | 1·19 | 7·46 | 20·80 | 29·45 |
| | *E'* | 0·35 | 1·59 | 6·62 | 21·29 | 29·85 |
| 1–69 | *O* | 0 | 4 | 27 | 99 | 130 |
| | *E* | 0 | 4 | 27 | 99 | 130 |
| | *E'* | 1·14 | 5·37 | 24·22 | 100·42 | 131·15 |

$\chi^2$ for 5 periods, total dose levels = $14 \cdot 99_E$, $14 \cdot 67_{E'}$, 4 d.f.

A further indication of goodness of fit can be illustrated from the relation

$$\log_e \log_e \frac{1}{1 - G} = \log_e b + k \log_e (t - w) \qquad (2)$$

obtained from (1) by taking natural logarithms twice. $G$ was estimated by the actuarially simulated number of tumours with a zero mortality standard population (Lee, 1970). Figures 1, 2 and 3 show the goodness of fit to the theoretical straight line relationship between $\log_e (t - w)$ and $\log_e \log_e (1/(1 - G))$ at either 4-week or 4 8-day intervals of $t$ as long as the number of tumours or carcinomas recorded up to time $t$ exceeds 2. For each point plus or minus one standard error bars are plotted. A separate line has been drawn for each dose level in which the total tumours or carcinomas exceeded 5 with data combined either over regimes or intakes. It seems clear that the fit to the Weibull distribution is very good indeed.

It is evident from inspection of Tables IV–VI and Fig. 1–3 that there is a very clear relationship between $\log_e b$ and dose. Assuming $k$ and $w$ are known we can, using methods described in the appendix, compute values of $\chi^2$ to test for differences in regime or intake, linear effect of dose, residual effects of dose and interaction between dose level and intake. It can be seen from Tables X, XI and XII that the main significant factor is the linear effect of dose and the

FIG. 1.

only other significant effect is a difference in intakes in the Gallaher Experiment. Thus the relationship

$$\log_e b_{ij} = u + a_i + \theta (\log \text{dose}_j) \qquad (3)$$

(where $u$ is the mean value of $\log_e b$, $a_i$ is the effect of the $i$th regime or intake, $j$ is the dose level and $\theta$ a constant) completely describes the data. The fitted values for this relationship are also given in Tables X–XII.

The fitted constant $\theta$ describes the dose-response relationship. In all three cases the value of $\theta$ is around 2 which means that increasing the dose by a factor of $C$ increases the incidence rate by a factor of $C^2$, for $b$ is in fact an incidence rate multiplying factor. The value of 2 is in fact just outside two standard

TABLE X.—*Variation of log b with Dose and Intake in Data of Table I. Battelle—Tumours*

| Effect | d.f. | Chi-squared | P |
|---|---|---|---|
| Intake . . . . . | 4 | 31·71 | < 0·005 |
| Dose linear . . . . | 1 | 846·35 | < 0·0001 |
| Dose residual . . . . | 2 | 0·36 | N.S. |
| Dose × Intake . . . | 12 | 9·53 | N.S. |

Model: $\log b_{ij} = U + a_i + \eta \log \text{dose}_j$; $U = -11{\cdot}351$; $\eta = 2{\cdot}040$, S.E. $\eta = 0{\cdot}092$; $a_1 = 0{\cdot}042$; $a_2 = 0{\cdot}377$; $a_3 = 0{\cdot}345$; $a_4 = -0{\cdot}353$; $a_5 = -0{\cdot}411$.

FIG. 2.

TABLE XI.—*Variation of log b with Dose and Regime in Data of Table II. Harrogate—Tumours*

| Effect | d.f. | Chi-squared | P |
|---|---|---|---|
| Regime . . . . . | 3 | 6·50 | < 0·1 |
| Dose linear . . . . | 1 | 537·16 | < 0·0001 |
| Dose residual . . . . | 2 | 0·73 | N.S. |
| Dose × Regime . . . | 9 | 11·40 | N.S. |

Model: $\log b_{ij} = U + a_i + \eta \log \text{dose}_j$; $U = -17{\cdot}622$; $\eta = 1{\cdot}782$, S.E. $\eta = 0{\cdot}090$; $a_1 = 0{\cdot}176$; $a_2 = 0{\cdot}069$; $a_3 = -0{\cdot}106$; $a_4 = -0{\cdot}139$.

TABLE XII.—*Variation of log b with Dose and Regime in Data of Table III. Harrogate—Infiltrating Carcinomas*

| Effect | d.f. | Chi-squared | P |
|---|---|---|---|
| Regime . . . . | 3 | 4·26 | N.S. |
| Dose linear . . . | 1 | 146·69 | <0·0001 |
| Dose residual . . | 1* | 0·73 | N.S. |
| Dose × Regime . . | 6 | 4·22 | N.S. |

Model: $\log b_{ij} = U + a_i + \theta$ (log dose$_j$); $U = -18{\cdot}767$; $\theta = 2{\cdot}304$, S.E. $\theta = 0{\cdot}239$; $a_1 = 0{\cdot}104$; $a_2 = 0{\cdot}233$; $a_3 = -0{\cdot}257$; $a_4 = -0{\cdot}080$.

* Only one degree of freedom as the 6 μg./week dose level was omitted because there were no infiltrating carcinomas. Assuming a linear relation the expected number of infiltrating carcinomas at this dose is approximately equal to one, which does not disprove the goodness of fit of the linear model.

FIG. 3.

deviations from the fitted value of $\theta$ for Harrogate Tumour Data whereas it is well inside it for both the other sets of data. It is certainly not possible for any integral value of $\theta$ but 2 to be consistent with the data so that if the multi-stage model of Armitage and Doll were true then benzopyrene affects two stages of the cancer process.

It remains to be shown that the fitted relationships subject to the dose response relationship of equation (3) actually fit the data adequately; Tables XIII–XV give the fitted values of $b$ in a similar manner to Tables IV–VI. Goodness of fit tests are given in Tables VII–IX where $E'$ is the expected number of tumours or carcinomas calculated in a similar manner to the $E$'s previously described (page 762) except that the $b$'s are those of Tables XIII–XV rather than those of Tables IV–VI.

TABLE XIII.—*Fitted Weibull Parameters to Data of Table I, Assuming the Linear Model Between log Incidence Rate and log Dose given in Table X. Battelle—Tumours*

$w = 14 \cdot 43$; $k = 2 \cdot 245$; values of $b \times 10^6$

| | Dose level µg./4 days | | | |
|---|---|---|---|---|
| Intake | 1 | 3 | 9 | 27 |
| 1 | 1·4 | 13·3 | 125·3 | 1178·4 |
| 2 | 2·0 | 18·6 | 175·2 | 1648·2 |
| 3 | 1·9 | 18·1 | 169·8 | 1596·8 |
| 4 | 1·0 | 9·0 | 84·5 | 794·6 |
| 5 | 0·9 | 8·5 | 79·7 | 749·4 |

TABLE XIV.—*Fitted Weibull Parameters to Data of Table II, Assuming the Linear Model Between log Incidence Rate and log Dose Given in Table XI. Harrogate—Tumours*

$w = 17 \cdot 70$; $k = 2 \cdot 954$; values of $b \times 10^7$

| | Dose level µg./week | | | |
|---|---|---|---|---|
| Dosing regime | 6 | 12 | 24 | 48 |
| 2 | 6·5 | 22·2 | 76·4 | 262·6 |
| 3S | 5·8 | 22·0 | 68·6 | 236·0 |
| 3F | 4·9 | 16·8 | 57·6 | 198·2 |
| 3½ | 4·7 | 16·2 | 55·7 | 191·6 |

TABLE XV.—*Fitted Weibull Parameters to Data of Table III, Assuming the Linear Model Between log Incidence Rate and log Dose Given in Table XII. Harrogate—Infiltrating Carcinomas*

$w = 22 \cdot 86$; $k = 2 \cdot 436$; values of $b \times 10^6$

| | Dose level µg./week | | | |
|---|---|---|---|---|
| Dosing regime | 6 | 12 | 24 | 48 |
| 2 | 0·5 | 2·4 | 11·9 | 58·7 |
| 3S | 0·6 | 2·7 | 13·5 | 66·8 |
| 3F | 0·4 | 1·7 | 8·3 | 40·9 |
| 3½ | 0·4 | 2·0 | 9·9 | 48·9 |

### DISCUSSION

It is clear that the model postulating the incidence rate of tumours from benzopyrene to be proportional both to (time $- w)^k$ and to $(\text{dose})^2$ is a very good fit to the data from our experiments. Similar analyses carried out in Harrogate on 7 experiments in which tobacco smoke condensate was painted at different dose levels showed a marked difference in the results. Here although over the dose range tested the log incidence rate increased in proportion to log dose the incidence rate–dose relationship was certainly not a quadratic one. Averaging the experiments the incidence rate was about proportional to $(\text{dose})^{1 \cdot 5}$.

Benzopyrene is well known to be present in tobacco smoke condensate in small quantities. However, the difference in dose response relationships means that benzopyrene cannot by itself be the cause of smoke condensate carcinogenesis. In any case calculations have shown that the amount of this carcinogen present in condensate is only sufficient to account for a small part—very approximately one-seventieth—of the carcinogenicity of stored condensate (T.R.C., 1970).

From the Armitage and Doll (1954) multistage hypothesis it seems possible that the quadratic response for benzopyrene is simply due to the carcinogen affecting two stages of the cancer process and that the 1·5th power response for condensate may well be an approximation from a mixture of a number of linear and quadratic carcinogenic responses. For, assumed that the carcinogens are non-interacting and that the linear part contributes $I_1$ to the incidence rate and the quadratic part $I_2$ for a given dose level $d$. Then at dose $2d$ the incidence rate will be $2I_1 + 4I_2$ and at dose $4d$ it will be $4I_1 + 16I_2$. As a compound carcinogen it will appear to be linear at very small doses and quadratic at very large doses but in the middle range, taking $I_1 = I_2$, successive doublings of dose multiply the rates by factors of 3 and 3·33. This sort of difference would be indistinguishable in a condensate experiment especially when one considers that for large doses, doubling the dose may well not double the effective dose due to piling up on the mouse's back.

In order to test dose–response relationships from multistage models further one would have to carry out experiments in which combinations of carcinogens at a wide range of dose levels are applied.

We thank Dr. R. F. Davies and his staff in Harrogate and Dr. W. Surber and Dr. A. Cerioli of the Battelle Institute, Geneva, for carrying out the experimental work.

### REFERENCES

ARMITAGE, P. AND DOLL, R.—(1954) *Br. J. Cancer*, **1**, 1.
COOK, P. J., DOLL, R. AND FELLINGHAM, S. A.—(1969) *Int. J. Cancer*, **4**, 93.
LEE, P. N.—(1970) *Biometrics*, **4**, 777.
PIKE, M. C.—(1966) *Biometrics*, **1**, 142.
RAO, C. R.—(1952) 'Advanced Statistical Methods in Biometric Research'. New York (Wiley).
T.R.C.—(1970) 'Review of Activities 1967–1969'.

### STATISTICAL APPENDIX

It can be shown (Pike, 1966) that the log likelihood function $L$ from a Weibull distribution for $r$ groups with known common $k$ and $w$ and unknown $b = b_i$ ($i = 1 \ldots r$) is given by

$$L = \sum_{i=1}^{r} s_i \log_e b_i - \sum_{i=1}^{r} b_i f_i \qquad (4)$$

where $s_i$ is the number of tumours in the $i$th group and $f_i$ is equal to

$$\sum_{x>w} (x - w)^k$$

in the $i$th group where $x$ for each animal is either its time of first tumour or its time of tumourless death.

Now consider an experiment in which each combination of $u = 1, 2 \ldots a$ treatments and $v = 1, 2 \ldots b$ dose levels $l_v$ are tested with $s_{uv}$ and $f_{uv}$ measured for each of the $ab$ combinations.

We can test the following hypotheses.

(1) No effect of treatment or dose

$$\log_e b_{uv} = u$$

(2) No effect of dose

$$\log_e b_{uv} = u + g_u$$

(3) No effect of treatment

$$\log_e b_{uv} = u + h_v$$

(4) No treatment dose interaction

$$\log_e b_{uv} = u + g_u + h_v$$

(5) All groups different

$$\log_e b_{uv} = u + c_{uv}$$

(6) No non-linear effect of dose or dose treatment interaction

$$\log_e b_{uv} = u + g_u + \theta\,(\log_e l_v)$$

In each model by substituting for log $b_{uv}$ in equation (4) and maximizing with respect to the different parameters we attain six different log likelihood maxima which we shall call $L_1$ through to $L_6$ respectively. $L_1$, $L_2$, $L_3$ and $L_5$ can be expressed explicitly in terms of the $s_{uv}$ and the $f_{uv}$ but $L_4$ and $L_6$ require an iterative procedure. Full mathematical details are too long to be given here but are available on request.

Using the Likelihood–Ratio Test we can set up a pseudo-analysis of variance table in the style of Rao (1952) with chi-squareds replacing mean squares. It is laid out as follows.

| Variation | D.F. | $\chi^2$ |
|---|---|---|
| Dose (ignoring treatment) | $b - 1$ | $-2(L_1 - L_3)$ |
| Treatment | $a - 1$ | $-2(L_3 - L_4)$ |
| Treatment (ignoring dose) | $a - 1$ | $-2(L_1 - L_2)$ |
| Dose | $b - 1$ | $-2(L_2 - L_4)$ |
| Dose Linear | 1 | $-2(L_2 - L_6)$ |
| Dose Residual | $b - 2$ | $-2(L_6 - L_4)$ |
| Dose $\times$ Treatment | $(a - 1)(b - 1)$ | $-2(L_4 - L_5)$ |